# Quantitative absorptive micro-sampling for decentralized monitoring of insulin-like growth factor 1

**DOI:** 10.3389/fbioe.2025.1648347

**Published:** 2025-09-15

**Authors:** Xiaogang Li, Zeqing Zhao, Shi Chen, Ye Guo, Hui Pan, Xiao Yang

**Affiliations:** ^1^ Biobank Facility, National Infrastructures for Translational Medicine, State Key Laboratory of Complex Severe and Rare Diseases, Peking Union Medical College Hospital, Chinese Academy of Medical Science and Peking Union Medical College, Beijing, China; ^2^ Department of Ultrasound Medicine, Peking Union Medical College Hospital, Chinese Academy of Medical Sciences and Peking Union Medical College, Beijing, China; ^3^ Key Laboratory of Endocrinology of National Health Commission, Department of Endocrinology, State Key Laboratory of Complex Severe and Rare Diseases, Peking Union Medical College Hospital, Chinese Academy of Medical Science and Peking Union Medical College, Beijing, China; ^4^ Department of Clinical Laboratory, State Key Laboratory of Complex Severe and Rare Diseases, Peking Union Medical College Hospital, Chinese Academy of Medical Science and Peking Union Medical College, Beijing, China; ^5^ Department of Ultrasound Medicine, Zhangzhou Municipal Hospital Affiliated to Fujian Medical University, Zhangzhou, Fujian, China

**Keywords:** growth hormone deficiency, IGF-1, quantitative absorptionmicro-sampling, mass spectrometry, analytical device

## Abstract

**Introduction:**

This study establishes a novel quantitative acoustic mass spectrometry (QAMS) methodology for insulin-like growth factor 1 (IGF-1) detection.

**Methods:**

Chromatographic separation utilized a Peptide C18 column (1.8 μm, 50 mm) with 0.1% formic acid/acetonitrile gradient elution, coupled to tandem mass spectrometry operated in scheduled multiple reaction monitoring (sMRM) mode.

**Results:**

The method demonstrated a lower limit of quantification (LOQ) of 10 ng/mL with linear dynamic range spanning 10-500 ng/mL. Comparative analysis of 74 paired plasma specimens revealed strong inter-matrix correlation with quantifiable bias.

**Discussion:**

These advancements position QAMS- as a robust tool for decentralized IGF-1 monitoring, particularly valuable in pediatric growth disorder studies and resource-limited settings. Longitudinal stability validation and isoform differentiation remain focal points for future optimization.

## 1 Introduction

Growth impairment disorders are defined by height measurements >2 standard deviations below age-, sex-, and ethnicity-matched population norms, combined with annualized growth velocity deficits. The pathophysiology involves dysregulation of the GH-IGF-1 axis: hypothalamic GHRH stimulates pulsatile GH secretion from the anterior pituitary, which subsequently induces hepatic IGF-1 synthesis. This endocrine cascade regulates longitudinal bone growth through epiphyseal plate chondrogenesis. Etiological factors include congenital mutations, acquired pituitary dysfunction, malnutrition, and chronic inflammatory conditions (LeRoith and yakar, 2007; Blum et al., 2018).

Among these, growth hormone deficiency (GHD) is one of the most common endocrine causes. Growth hormone (GH) is an important hormone secreted by the anterior pituitary gland, which not only promotes growth but also regulates material metabolism. GH secretion exhibits ultradian rhythmicity, with peak amplitudes occurring during slow-wave sleep. Single serum GH measurements exhibit poor diagnostic sensitivity, necessitating provocative testing. While 24-h GH profiling improves specificity for GH neurosecretory dysfunction, its clinical utility is limited by procedural complexity (6–8 venipunctures/24 h) and low patient compliance (Giacomozzi et al., 2023; Melmed, 2019).

IGF-1 is mainly produced by hepatocytes and primarily binds with specific IGF binding protein-3 (IGFBP-3) to form a stable ternary complex. Serum concentrations rise progressively from infancy to pubertal peak, making IGF-1 quantification a cornerstone for GHD screening in children >5 years. Current chemiluminescence immunoassay (CLIA) suffer from antibody cross-reactivity. Liquid chromatography coupled to tandem mass spectrometry (LC-MS/MS) has become a more frequently applied clinical detection method in recent years, offering high specificity and sensitivity to detect multiple proteins simultaneously (Melmed, 2019).

Well-preserved blood samples play a critical role in the detection accuracy of IGF-1. However, existing IGF-1 detection technologies focus more on the laboratory phase and neglect the collection and preservation of blood samples. Since IGF-1 in the blood is susceptible to degradation or denaturation due to factors such as temperature, light exposure, and pH levels, it can lead to inconsistent detection results with the actual values, preventing accurate measurement of IGF-1 concentrations in the human body (Delahaye et al., 2021; Spooner et al., 2020; Guerra Valero et al., 2022; Wickremsinhe et al., 2022; Verhaeghe et al., 2020; Lee et al., 2023).

This project develops a method for detecting IGF-1 based on quantitative absorption micro-sampling (QAMS), which reduces the impact of the storage environment and the volume (10 μL–30 μL) of blood samples. The dried blood spot samples are pre-treated to extract the supernatant sample containing the IGF-1 internal standard working solution, without using surfactants to dissociate and release IGF-1. The supernatant sample is purified by SPE and detected using LC-MS/MS. The method requires only a small amount of blood sample, significantly reducing the amount of blood collection in existing detection methods.

## 2 Methods and material

### 2.1 Reagents and chemicals

VitaPad,10 μL, BaiQu (Shanghai, China); Oasis MAX 96-well µElution Plate, 2 mg Sorbent per Well, 30 μm, Waters (CT, United States), QuanRecovery with MaxPeak, 700 µL Plates, Waters (CT, United States), 96-Well Plate Low adsorption-sample plate, 2 mL, Shimadzu (Kyoto, Japan); Protein LoBind^®^ Tubes, Eppendorf (Hambourg, Germany). Bovine serum albumin and Phosphate buffer tablets, Sigma - Aldrich (MO, United States). SeraCon™II Double Stripped Delipidated Narmal Human Plasma (IGF-1 Free Human Plasma), SeraCare (Maryland, United States). IGF-1 and Fully ^15^N-labeled IGF-I, ProSpec (NessZiona, Israel). National Institute of Standards and Technology Standard Reference Material 2,926 Recombinant Human Insulin-like Growth Factor 1 (Frozen) (Washington, United States).

### 2.2 QAMS dry blood spot sample preparation

Samples were collected from 74 healthy subjects in Beijing, including males and females, individuals aged from 2 to 60. Samples once collected were immediately anonymized and after use immediately destroyed. The subjects or their guardians were informed about the aim of our study and gave their consent for the use of blood samples for research.

Pull out the capillary cap with the quantitative capillary and tip of the quantitative capillary into the blood drop until fully filled. Then place the capillary cap back, the tip of the quantitative capillary comes into contact with the sample carrier. The sample is transferred from the quantitative capillary tube to the sample carrier and observed through the transparent observation window until all samples in the quantitative capillary are transferred to the sample carrier. Samples were dried within vitapad by desiccant.

### 2.3 Pretreatment of dry blood spot sample

The sample pretreatment protocol consisted of three sequential phases: preparation of internal standard solutions, extraction of analytes, and solid-phase extraction (SPE) purification, as detailed below.

#### 2.3.1 Internal standard preparation

A stock solution of IGF-I internal standard (2 μg/mL in 5% bovine serum albumin, BSA) underwent 50-fold dilution with deionized water to achieve a working concentration of 40 ng/mL.

#### 2.3.2 Sample extraction procedure

A 10 μL dried blood spot disc was transferred to a 1.5 mL polypropylene centrifuge tube. The extraction process commenced with addition of 300 μL internal standard working solution followed by 30-min ultrasonication. Subsequently, 150 μL of acetonitrile containing 5% acetic acid (v/v) was added for protein precipitation. After vortex-mixing (900 rpm, 10 min) with 300 μL 5% ammonium hydroxide (v/v), the mixture underwent centrifugation at 4,000 × g for 5 min to collect supernatant.

#### 2.3.3 SPE purification protocol

The SPE cartridge was sequentially preconditioned with 200 μL methanol and 200 μL 5% ammonium hydroxide (v/v). The supernatant was loaded onto the activated cartridge and subjected to two-step washing: first with 200 μL 5% ammonium hydroxide (v/v), followed by 200 μL methanol aqueous solution (5% methanol, 1% acetic acid, v/v). Analytes were eluted using sequential 40 μL aliquots of 60% methanol containing 5% acetic acid (v/v) and deionized water.

### 2.4 Detection of insulin-like growth factor content

#### 2.4.1 Configuration of liquid chromatography-mass spectrometry (LC/MS)

Chromatographic separation was achieved using a reversed-phase C18 column (1.8 μm particle size, 50 mm length) maintained at 25 °C. A binary mobile phase system comprising phase A (0.1% formic acid in deionized water, v/v) and phase B (neat acetonitrile) was delivered at 0.4 mL/min through a nonlinear gradient elution profile over 5 min, as detailed in Table 1. Sample introduction employed a fixed 10 μL injection volume with a 1.0 min needle wash cycle between runs.

**TABLE 1:** Elution gradient.

Time (min)	Flow rate(mL/min)	Mobile phase A (%)	Mobile phase B (%)
0	0.4	98	2
0.50	0.4	98	2
1.50	0.4	80	20
3.00	0.4	50	50
3.20	0.4	10	90
4.00	0.4	10	90
4.01	0.4	98	2
5	4.0	98	2

Electrospray ionization (ESI) in positive mode was optimized with the following critical parameters: capillary voltage maintained at 1.0 kV, desolvation gas heated to 600 °C (N_2_ flow: 1100 L/h). The source offset voltage was calibrated to 50 V to balance sensitivity and in-source fragmentation. Multiple reaction monitoring (MRM) transitions were configured with collision energies ranging 27–35 eV with specific precursor-product ion pairs enumerated in Table 2.

**TABLE 2:** MRM parameter data.

Name	Parent Ion (m/z)	Daughter Ion (m/z)	Cone voltage(V)	Collision energy(V)
IGF-1	957	473.3	50	35
IGF-1	957	1,175.6	50	27
IGF-1	957	1,196.9	50	27
IGF-1	1,093.5	473.3	50	35
IGF-1	1,093.5	1,196.9	50	35
N15 IGF internal standard	968.5	1,189.5	30	35
N15 IGF internal standard	1,106.8	1,211.2	30	35

#### 2.4.2 Calibration curve preparation

A concentrated stock solution (designated 20RL-H, 20× working concentration) was reconstituted in a ternary solvent system comprising 10% (v/v) acetic acid, 30% (v/v) methanol, and 60% (v/v) deionized water. The 20RL-H stock underwent six-step geometric dilution using the initial solvent matrix, producing intermediate calibration points 20*C1, 20*C2, 20*C3, 20*C4, 20*C5, 20*C6. Bovine serum albumin-enriched diluent (5% BSA, w/v; 0.1% ProClin™300 antimicrobial agent, v/v) served as biological matrix simulator. A secondary 20-fold dilution of each intermediate solution (20*C1–20*C6) in this proteinaceous medium yielded final calibration standards C_1_–C_6_ spanning10–500 ng/mL (Table 3).

**TABLE 3:** Calibration standard C1-C6 concentration data.

Calibration standard C1-C6 concentration data	20*C1 (ng/mL)	20*C2 (ng/mL)	20*C3 (ng/mL)	20*C4 (ng/mL)	20*C5 (ng/mL)	20*C6 (ng/mL)
Standard Concentration	200	500	1,000	2000	4,000	10,000
Diluted Concentration (20X)	10	25	50	100	200	500

### 2.5 Detection of SPE-Purified blood samples

SPE-purified samples were analyzed via hyphenated liquid chromatography-tandem mass spectrometry (LC-MS/MS) with scheduled multiple reaction monitoring (sMRM). Quantification was achieved through internal calibration using six-point matrix-matched standards (C1–C6). Linearity was verified by measuring the linearity reference standards. Linear reference standards (RL1–7) were prepared by spiking IGF-1 free human plasma with target concentrations ranging from 10 to 1,000 ng/mL, measuring three technical replicates per linearity level. System suitability criteria mandated a linear regression coefficient (R^2^) ≥0.99 across the 10–1,000 ng/mL dynamic range, coupled with ≤15% Coefficient of variation (CV) The accuracy of the method was verified through spike recovery studies, where all measured values fell within the validated acceptance range of 85%–115% for IGF-1 quantification. The intraday precision was validated by measuring the coefficient of variation (CV) of 10 replicats at two different concentration levels. The inter-operator precision was validated by measuring the CV of 2 operators, each operator analyzed 5 replicates for two different concentration levels. For each concentration level, the CV of 10 replicate measurements should not exceed 15%.

#### 2.5.1 Inter-participant sampling study

To evaluate the performance of vitapad for IGF-1 measurement, a prospective sampling study was conducted involving 3 healthy participants (S1, S2, S3). The protocol specified consecutive 3-day sampling for each participant, with 3 replicate samples collected per day. The overall coefficient of variation (CV) should ≤15%.

#### 2.5.2 Stability profiling protocol

Thermal degradation kinetics were evaluated using human whole blood aliquots (n = 3 per condition) collected on VitaPad matrix (10 μL spotting volume). Accelerated stability studies employed 72-h storage at 37 °C (physiological stress) and 50 °C (extreme degradation), benchmarked against −80 °C cryopreserved controls. Real time sample stability was evaluated using human whole blood aliquots (n = 3 per condition) collected on VitaPad matrix (10 μL spotting volume). Real time stability studies employed 3/7 days at 25 °C and 2/4 weeks at 4 °C, benchmarked against −80 °C cryopreserved controls.

Post-treatment samples underwent identical SPE-LC/MS workflows to quantify IGF-1 recovery rates, with degradation calculated via [(Treated sample concentration)/(Control concentration)]×100%.

#### 2.5.3 Comparative study

**FIGURE 1:**
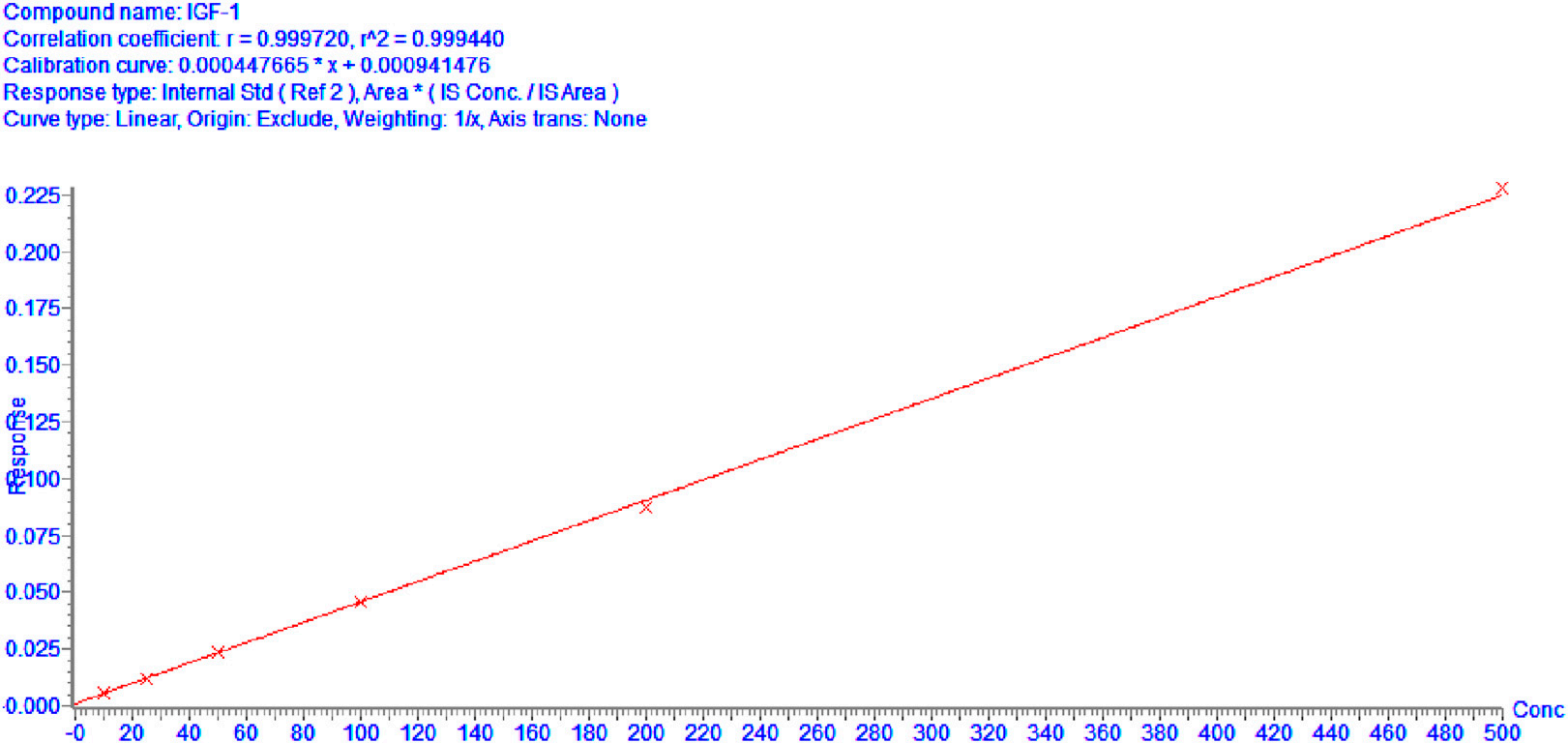
Calibration curves of IGF-1 (10–500 ng/mL).

The minimum sample size requirement calculated by G*Power (version 3.1.9.7) is 42 when estimating the following scenarios (power = 95%) and significance level (α = 0.05). The correlation analysis revealed a strong linear relationship between the two sample types, indicating high correlation despite the observed bias (Figure 1). The power of 74 samples calculated as 0.998 with an effect size of 0.59 and setting β/α ratio as 1. Methodological equivalence was assessed through parallel analysis of 74 paired clinical specimens (10 μL QAMS vs. 10 μL plasma, matched donor sets).

Healthy subjects with normal physical examination findings, and stable vital signs, without history of chronic diseases or acute illnesses within the past 4 weeks. Including males and females, individuals aged from 2 to 60. Samples once collected were immediately anonymized and after use immediately destroyed. The subjects or their guardians were informed about the aim of our study and gave their consent for the use of blood samples for research.

Subsequently, correlation analysis was conducted to investigate the relationship between these two sample types.

#### 2.5.4 Hematocrit (HCT) effect study

Centrifuge sheep whole blood (without human IGF-1 in the matrix) to obtain plasma and red blood cells, measure HCT values using a hemoglobin analyzer, and reconstruct whole blood with different HCT values by adding different proportions of plasma and red blood cells and gently mixing them evenly. Adjust HCT to 40% ± 2%, 50% ± 2%, and 60% ± 2%. Then, low, medium, and high (50, 200, 500 ng/mL) concentrations of IGF-1 were added, gently mixed, and QAMS samples were prepared. The accuracy was verified by the measurement results, and 85%–115% met the requirements.

## 3 Results

### 3.1 Pretreatment optimization

This optimized workflow eliminated requirements for surfactant additives (e.g., CHAPS, TFE, SDS) traditionally employed for IGF-1 dissociation, thereby minimizing potential mass spectrometry contamination risks. The simplified reagent composition enhanced analytical system compatibility while maintaining efficient target recovery.

### 3.2 IGF-1 quantification and methodological validation

Calibration curves demonstrated exceptional linear performance across 10–500 ng/mL.

As shown in Figure 1, when curve was fitted using linear regression with origin exclusion and 1/x weighting. The linear regression equation for IGF-1 was y = 0.000449672x + 0.00041476 with a correlation coefficient (R^2 = 0.9994), indicating excellent linearity over the concentration range of 10–500 ng/mL.

Table 4 presents the validation results for linearity, evaluated using seven linear reference standards (RL1–RL7) with target concentrations ranging from 10 to 1,000 ng/mL. For each reference standard, the measured mean concentrations (9.73–1,012.24 ng/mL) showed good agreement with the theoretical values, as indicated by relative errors (RE) ranging from −2.67% to 1.64%, all within the acceptable range of ≤±15%. Additionally, the coefficient of variation (CV) for each concentration level was low (0.43%–5.74%), demonstrating high precision of the measurements. These results confirm that the analytical method exhibits excellent linearity across the tested concentration range of 10–1,000 ng/mL.

**TABLE 4:** Linear reference standard detection results.

RL	Standard Conc. (ng/mL)	Mean Conc (ng/mL)	CV (%)	RE (%)
RL1	10	9.73	5.74%	−2.67%
RL2	25	25.58	1.53%	2.33%
RL3	50	49.60	2.37%	−0.81%
RL4	100	99.87	3.05%	−0.13%
RL5	200	203.28	2.26%	1.64%
RL6	500	508.09	1.67%	1.62%
RL7	1,000	1,012.24	0.43%	1.22%

To further verify the detection limit of this detection method, reference samples of 10 ng/mL, 5 ng/mL, and 2 ng/mL were rigorously evaluated, and the results are shown in Table 5.

**TABLE 5:** Detection limit detection results.

Standard Conc. (ng/mL)	Mean Conc (ng/mL)	CV (%)	RE (%)
10	9.73	5.74%	−2.67%
5	5.37	17.10%	7.36%
2	1.58	23.75%	−21.18%

Among them, the biases and CVs of 10 ng/mL and 5 ng/mL meet the quantitative limit acceptance standards (bias≤15%, and CV ≤ 15%); while the bias or CV at 2 ng/mL and 5 ng/mL exceeded the acceptance criteria, which may be related to the insufficient signal-to-noise ratio and baseline noise interference. Therefore, the LOQ of this embodiment is determined to be 10 ng/mL.

### 3.3 IGF-1 spiked sample recovery

To evaluate the accuracy and reliability of the method, spike recovery experiments for IGF-1 were conducted. As shown in Table 6 spiked samples at low (25 ng/mL), medium (50 ng/mL), and high (200 ng/mL) concentration levels were analyzed.

**TABLE 6:** Spiked sample recovery.

Spiked sample recovery	Mean Conc. (ng/mL)	CV (%)	Spiked Conc. (ng/mL)	Recovery rate (%)
RR S1_0	58.12	4.00%	—	—
RR S1_L	81.31	0.90%	25	92.75%
RR S1_M	114.17	5.50%	50	112.09%
RR S1_H	244.93	5.20%	200	93.40%
RR S2_0	79.69	2.80%	—	—
RR S2_L	107.99	9.50%	25	113.21%
RR S2_M	134.27	7.70%	50	109.15%
RR S2_H	273.27	3.40%	200	96.79%

As summarized in Table 6, the background concentrations of IGF-1 in unspiked samples (S1_0 and S2_0) were 58.12 ng/mL and 79.69 ng/mL, respectively. The spike recovery results (92.75%–113.21%) met the predefined acceptance criteria (85%–115%) for method validation.

### 3.4 IGF-1 precision

The intraday and inter-operator precision was conducted.

The CVs under all conditions is not greater than 15%, (Tables 7, 8) which demonstrates the good repeatability of the method.

**TABLE 7:** Intraday precision.

Intraday precision	Measured Conc. (ng/mL)	CV (%)
L-1	56.95	5.8%
L-2	59.46	
L-3	52.43	
L-4	50.41	
L-5	56.73	
L-6	56.39	
L-7	53.48	
L-8	51.03	
L-9	54.14	
L-10	50.53	
H-1	189.36	3.9%
H-2	173.32	
H-3	186.45	
H-4	194.26	
H-5	184.00	
H-6	189.15	
H-7	181.26	
H-8	176.28	
H-9	176.72	
H-10	175.08	

**TABLE 8:** Inter-operator precision.

Inter-operator precision	Measured Conc. (ng/mL)	CV (%)
L- Operator1	58.76	2.82%
	62.27	
	60.50	
	58.38	
	62.93	
L- Operator2	58.44	
	59.45	
	60.57	
	61.55	
	58.46	
H- Operator1	233.18	4.70%
	233.71	
	233.35	
	226.15	
	200.51	
I- Operator2	224.90	
	236.19	
	232.41	
	238.01	
	230.85	

### 3.5 Detection performance of samples

Above table presents the IGF-1 measurement results using vitapad, with three consecutive daily samples (3 replicates per day) collected from 3 participants (S1, S2, S3). For S1, S2 and S3 with an overall coefficient of variation (CV) of 11.8%, 8.3% and 13.5%. These results indicate consistent intra-participant reproducibility across the 3-day sampling period, supporting the reliability (Table 9) of vitapad for IGF-1 measurement.

**TABLE 9:** Inter-Participant Sampling results.

Inter-participant sampling results	Measured Conc. (ng/mL)	Mean Conc. (ng/mL)	CV (%)
S1day1	106.90	101.71	11.8%
S1day1	106.88		
S1day1	91.35		
S1day2	114.49	109.00	
S1day2	114.47		
S1day2	98.04		
S1day3	130.67	124.58	
S1day3	130.65		
S1day3	112.41		
S2day1	184.40	182.12	8.3%
S2day1	180.81		
S2day1	181.14		
S2day2	196.51	194.09	
S2day2	192.71		
S2day2	193.06		
S2day3	221.71	219.03	
S2day3	217.49		
S2day3	217.88		
S3day1	52.18	47.38	13.5%
S3day1	43.77		
S3day1	46.21		
S3day2	56.58	51.50	
S3day2	47.68		
S3day2	50.26		
S3day3	66.39	60.76	
S3day3	56.51		
S3day3	59.38		

#### 3.5.1 Thermal stability assessment

Although both samples S1 and S2 exhibited concentration variations when stored at 37 °C and 50 °C for 3 days compared to the reference stored at −80 °C, these changes did not indicate significant degradation. The percentage changes in concentration were relatively small and within an acceptable range. This suggests that the samples have a certain degree of stability under the tested conditions, and short-term storage at these temperatures (Table 10) may not lead to substantial degradation of the samples. However, continuous monitoring and further studies over longer time periods would be beneficial to comprehensively assess the long-term stability of the samples.

**TABLE 10:** Thermal Stability results.

Condition	S1 (ng/mL)	S1 (RE, %)	S2 (ng/mL)	S2 (RE, %)
Ref (−80 °C)	87.61	—	158.85	—
37_3d	77.96	−11.02%	166.80	5.01%
50_3d	84.53	−3.52%	170.23	7.17%

#### 3.5.2 Sample stability assessment

All bias results are not greater than 15%. Thus IGF-1 can be stored at room temperature for no less than 7 days and refrigerated for no less than 4 weeks in QAMS (Table 11).

**TABLE 11:** Sample Stability results.

Condition	S3 (ng/mL)	S3 (RE, %)	S4 (ng/mL)	S4 (RE, %)
Ref(-80 °C)	61.54	—	244.41	—
25 °C_3 Day	58.47	−5.0%	251.99	3.1%
25 °C_7 Day	57.27	−6.9%	248.08	1.5%
4 °C_2 Week	62.15	1.0%	237.79	−2.7%
4 °C_4 Week	62.52	1.6%	249.20	2.0%

#### 3.5.3 Comparative study

**FIGURE 2:**
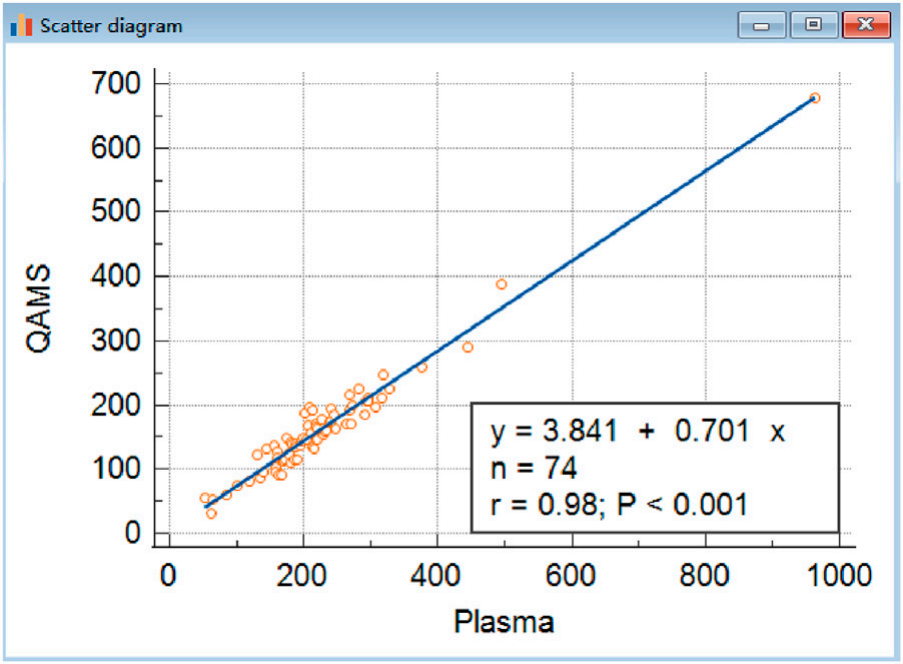
Comparison of IGF-I in QAMS and plasma. The correlations of venous blood plasma and corresponding dried capillary blood spot IGF-I (y = 3.8406 + 0.7013x,R^2^ was 0.9572, n = 74, P < 0.001) are shown. Approximately, the concentration of IGF-1 in QAMS sample divided by 0.7 equals its concentration in plasma.

The correlation analysis revealed a strong linear relationship between the two sample types, indicating high correlation despite the observed bias (Figure 2). The power of 74 samples calculated as 0.998 with an effect size of 0.59 and setting β/α ratio as 1.

#### 3.5.4 Hematocrit (HCT) effect study

Hematocrit effect was evaluated by assessing the accuracy and precision of samples spiked with analytes at three concentrations (50, 200, and 500 ng/mL) across three HCT levels (40%, 50%, and 60%) (Table 12).

**TABLE 12:** Hematocrit (HCT) Effect results.

HCT-spiked Conc. (ng/mL)	Mean Conc. (ng/mL)	CV (%)	Accuracy (%)
40%-50	43.17	2.19%	86.35%
40%-200	204.07	2.97%	102.04%
40%-500	546.24	5.68%	109.25%
50%-50	53.66	6.23%	107.32%
50%-200	201.14	2.92%	100.57%
50%-500	450.94	6.22%	90.19%
60%-50	56.50	4.04%	112.99%
60%-200	200.38	1.21%	100.19%
60%-500	517.17	3.73%	103.43%

Accuracy values varied from 86.35% to 112.99%, with most samples falling within 85–115%. The results suggest minimal hematocrit-dependent interference across the tested HCT, ranges.

## 4 Conclusion

The insulin-like growth factor (IGF-1) detection method based on quantitative acoustic mass spectrometry (QAMS) only requires the collection of a small amount of blood samples that are dried to form DBS samples. The drying and storage are completed in an environment relatively isolated from the outside world, reducing the interference from the external environment on the samples and enhancing their stability. The methodology’s enhanced sensitivity (LOQ = 10 ng/mL) enables precise quantification (spiked recovery between 92.75% and 113.21%) while maintaining linear dynamic range across three orders of magnitude (10–1,000 ng/mL). The less invasive sampling method reduces patient burden and improves compliance, thereby enabling more frequent monitoring and denser data collection. This facilitates the capture of dynamic physiological changes and supports early detection and intervention of growth abnormalities. This feature makes it particularly suitable for scenarios such as: monitoring of growth hormone therapy; large-scale growth development screening; long-term follow-up management.

The IGF-1 detection method using QAMS has brought new opportunities in IGF-1 research and applications, yet there’s room for improvement. Although it shows feasibility and advantages in sample collection and short-term stability, long-term stability studies are vital. Future work should monitor IGF-1 changes in dried blood spots stored differently for years to enhance the reliability of QAMS samples for retrospective and longitudinal studies, especially for pediatric growth disorders (ICH E11, 2017; Maus et al., 2020; Motorykin et al., 2021; Moncrieffe et al., 2020; To et al., 2021).

Moreover, the bias between QAMS dried blood spots and plasma samples despite their high correlation requires further exploration. Understanding its causes could lead to better calibration and preparation techniques. Research could examine the influence of blood components and physiological states on IGF-1 extraction and accuracy in QAMS samples. Larger and more diverse comparative studies would also help clarify result variability and generalizability (Guthrie et al., 2020; Mohammed-Ali et al., 2022; Ibba et al., 2020; Simstich et al., 2023; Ezra et al., 2023). Overall, continuous research is needed to fulfill the potential of QAMS in IGF-1 research and practice.

## Data Availability

The raw data supporting the conclusions of this article will be made available by the authors, without undue reservation.

## References

[B1] Blum W. F. Alherbish A. Alsagheir A. El Awwa A. Kaplan W. Koledova E. (2018). The growth hormone-insulin-like growth factor-I axis in the diagno sis and treatment of growth disorders. Endocr. Connect. 7 (6), R212–R222. 10.1530/ec-18-0099 29724795 PMC5987361

[B2] Delahaye L. Veenhof H. Koch B. C. P. Alffenaar J. C. Linden R. Stove C. (2021). Alternative sampling devices to collect dried blood microsamples: state-of-the-art. Ther. Drug Monit. 43 (3), 310–321. 10.1097/ftd.0000000000000864 33470777

[B3] Ezra S. Winstone T. M. L. Singh R. Orton D. J. (2023). Agreement of LC-MS assays for IGF-1 traceable to NIST and WHO standards permits harmonization of reference intervals between laboratories. Clin. Biochem. 116, 75–78. 10.1016/j.clinbiochem.2023.04.002 37031902

[B4] Giacomozzi C. Martin A. Fernández M. C. Gutiérrez M. Iascone M. Domené H. M. (2023). Novel insulin-like growth factor 1 gene mutation: broadening of the phenotype and implications for insulin resistance. J. Clin. Endocrinol. Metab. 108 (6), 1355–1369. 10.1210/clinem/dgac738 36546343

[B5] Guerra Valero Y. Dorofaeff T. Parker L. Coulthard M. G. Sparkes L. Lipman J. (2022). Microsampling to support pharmacokinetic clinical studies in pediatrics. Pediatr. Res. 91 (6), 1557–1561. 10.1038/s41390-021-01586-4 34023854

[B6] Guthrie H. Honig L. S. Lin H. Sink K. M. Blondeau K. Quartino A. (2020). Safety, tolerability, and pharmacokinetics of crenezumab in patients with mild-to-moderate Alzheimer’s disease treated with escalating doses for up to 133 weeks. J. Alzheimers Dis. 76 (3), 967–979. 10.3233/jad-200134 32568196 PMC7505005

[B7] Ibba A. Corrias F. Guzzetti C. Casula L. Salerno M. di Iorgi N. (2020). IGF1 for the diagnosis of growth hormone deficiency in children and adolescents: a reappraisal. Endocr. Connect. 9 (11), 1095–1102. 10.1530/ec-20-0347 33112822 PMC7774770

[B8] ICH E11 (2017). Clinical investigation of medicinal products in the pediatric population (R1). Available online at: https://database.ich.org/sites/default/files/E11R1Addendum.pdf. 12362934

[B9] Lee J. Cradic K. Singh R. Jones J. Li J. (2023). Discordance of insulin-like growth factor-1 results and interpretation on four different platforms. Clin. Chim. Acta. 539, 130–133. 10.1016/j.cca.2022.11.034 36528048

[B10] LeRoith D. yakar S. (2007). Mechanisms of disease: metabolic effects of growth hormone and insulin-like growth factor 1. Nat. Clin. Pract. Endocrinol. Metab. 3 (3), 302–310. 10.1038/ncpendmet0427 17315038

[B11] Maus A. Kemp J. Milosevic D. Renuse S. Pandey A. Singh R. J. (2020). Center of mass cal culation in combination with MS/MS allows robust identification of single amino acid polymorphisms in clinical measurements of insulin-like growth factor-1. J. Proteome Res. 19 (1), 186–193. 10.1021/acs.jproteome.9b00494 31736316

[B12] Melmed S. (2019). Pathogenesis and diagnosis of growth hor mone deficiency in adults. N. Engl. J. Med. 380 (26), 2551–2562. 10.1056/nejmra1817346 31242363

[B13] Mohammed-Ali Z. Delaney S. Singh R. Leung F. Taher J. Goguen J. (2022). Bias in IGF-1 concentrations and interpretation across three different clinical laboratory assays. Clin. Biochem. 108, 14–19. 10.1016/j.clinbiochem.2022.06.009 35772500

[B14] Moncrieffe D. Cox H. D. Carletta S. Becker J. O. Thomas A. Eichner D. (2020). Inter-laboratory agreement of insulin-like growth factor 1 concentrations measured intact by mass spectrometry. Clin. Chem. 66 (4), 579–586. 10.1093/clinchem/hvaa043 32232452

[B15] Motorykin I. Li H. Clarke N. J. McPhaul M. J. Wu Z. (2021). Isotopic peak index, relative retention time, and tandem MS for automated high throughput IGF-1 variants identification in a clini cal laboratory. Anal. Chem. 93 (34), 11836–11842. 10.1021/acs.analchem.1c02566 34461729

[B16] Simstich S. Züllig T. D’Aurizio F. Biasotto A. Colao A. Isidori A. M. (2023). The impact of dif ferent calibration matrices on the determination of insulin-like growth factor 1 by high-resolution-LC-MS in acromegalic and growth hormone deficient patients. Clin. Biochem. 114, 95–102. 10.1016/j.clinbiochem.2023.02.008 36849049

[B17] Spooner N. Anderson M. Wickremsinhe E. R. (2020). Patient-centric sampling special focus issue. Bioanalysis 12 (13), 867–868. 10.4155/bio-2020-0176 32772899

[B18] To M. Raizman J. E. Goudreau B. L. Higgins T. Brun M. Tsui A. K. (2021). Centralization of multisite reagent lot-to-lot validation for ortho Clinical Vitros chemistry instruments. Clin. Biochem. 97, 62–66. 10.1016/j.clinbiochem.2021.07.017 34343576

[B19] Verhaeghe T. Meulder M. Hillewaert V. Dillen L. Stieltjes H. (2020). Capillary microsampling in clinical studies: opportunities and challenges in two case studies. Bioanalysis 12 (13), 905–918. 10.4155/bio-2020-0054 32628039

[B20] Wickremsinhe E. Anderson M. Spooner N. (2022). Opportunities for improving clinical decisions with patient centric remote blood sampling approaches. Appl. Clin. Trials 31 (4), 20–22. Available online at: https://www.appliedclinicaltrialsonline.com/view/opportunities-for-improving-clinical-decisions-with-patient-centric-remote-blood-sampling-approaches.

